# The Localisation of Hæmoptysis

**Published:** 1921-01-22

**Authors:** 


### The Localisation of Hæmoptysis.

This is frequently a subject of interest, since even clinically tuberculous disease of the lungs is in the great majority of instances bilateral. Moreover, it is by no means always the case that when hæmoptysis occurs the blood comes from the side of the severer, or of the more extensive, disease. Of course the usual methods of treatment of the complication in question do not call for an exact topographical diagnosis. For instance, the icebag, which it was customary to lay on the side supposed to be the site of hæmorrhage, is of quite doubtful utility. Even the strapping of that side, with the idea of restraining its movement, may be responsible for hindering natural expulsive efforts to get rid of blood clot. But now that artificial pneumothorax therapy offers such a valuable means against dangerous or obstinate hæmoptysis, any additional diagnostic points for the ascertaining of which lung is bleeding become of great value. And recently attention has been drawn to one, or rather re-drawn.

Signs from the Phrenic Nerve.

As far back as the 'seventies de Mussy described points of tenderness to pressure at various points in the course of the phrenic nerve on the side on which active pulmonary tuberculosis was present. He and his school enumerated five such points from above downwards. First, between the two heads of the sterno-mastoid, that is at the place where the nerve crosses the scalenus anticus; secondly, along the sternal border in the first or second intercostal space; thirdly, the "bouton diaphragmatique," at the point of intersection of imaginary lines continuing the tenth rib and the parasternal line; fourthly, the place of insertion of the diaphragm at the base of the thorax—Huchard's "demi-ceinture douloureuse"; lastly, the region of the cervical plexus and of the spines of the cervical vertebræ, from radiation of sensitiveness along the paths of nerves having a common origin with the phrenic; the phenomenon here being advanced as analogous with the well-known shoulder pain and pain in the supraclavicular region of the side affected. Neumann has lately taken up the subject, and put forward a *Casuistik* to show that the tenderness in the above places really due to lung tubercle may lead to mistaken diagnosis of local disease, such as duodenal ulcer. He urges, too, that not only the side of active tuberculosis may be thus ascertained, with, of course, the special application to the localisation of hæmoptysis, but also the diagnosis of occult phthisis is facilitated.

Tender Spots after Hæmoptysis.

He traces the origin of these tender spots after hæmoptysis (only a considerable bleeding causes them) to small infections in the lung base from foci of aspiration pneumonia. These, being in contact with the diaphragm or the mediastinum, set up irritation of the phrenic nerve. They appear about the fourth day after the hæmorrhage and may soon pass off. They can be set up by the focal reaction to a diagnostic injection of tuberculin. The only source of fallacy mentioned seems to be diaphragmatic pleurisy in a sub-chronic pleurisy following a pneumonic condition of the lung.

The potential importance of these observations does not need stressing, and no doubt tuberculosis clinicians will give them a trial in practice.

